# Cytosolic Distribution of Cd^2+^, Cu^2+^, and Zn^2+^ Among Metallothionein-Like Proteins in Lobster Hepatopancreas

**DOI:** 10.1155/MBD.1994.525

**Published:** 1994

**Authors:** Joseph Schultz, Zhiwu Zhu, David H. Petering, C. Frank Shaw III

**Affiliations:** 1 Department of Chemistry University of Wisconsin-Milwaukee Milwaukee WI 53201-0413 USA


					CYTOSOLIC
AMONG
IN
DISTRIBUTION OF Cd2., Cu*, AND Zn2*
METALLOTHIONEIN-LIKE PROTEINS
LOBSTER HEPATOPANCREAS
Joseph Schultz, Zhiwu Zhu David H. Petering, and C. Frank Shaw III
Department of Chemistry, University of Wisconsin-Milwaukee, Milwaukee, Wl-53201-0413, USA
Zinc and Cu metabolism in crustacea changes dramatically during the molt cycle. Metallothionein
(MT) and MT-like proteins are implicated in the handling of both metal ions as well as in the
detoxification of Cd. Distribution of Cd2 /
Cu/
and Zn2 /
in cytosol and among MT-like species
has been determined. Lobsters were treated to induce MT as in mammals with dexamethasone
(dex) or stress from the surgical removal of a small walking toe both with and without a subsequent
injection of CdCI2. Untreated tissue contains only Cu-MTII and no other significant amounts of
metal-bound MTs. Exposure to dex or surgery alone causes no change in the MTs. Exposure to
Cd2/
retains the Cu-MTII and produces little Cd-MTI,II. When dex or surgery is combined with
Cd2 /
injection, Cu-MTII is unaffected and large quantities of Cd-MTI,II are made. Preliminary
results indicate that dex or surgery induces the synthesis of apo-MT in the hepato-pancreas which
serves as a site for binding cadmium.
Supported by NIH grants ES-04026 and ES-04184.
525

				

